# Pediatric Extra-Renal Nephroblastoma (Wilms’ Tumor): A Systematic Case-Based Review

**DOI:** 10.3390/cancers15092563

**Published:** 2023-04-29

**Authors:** Akzhol Karim, Kundyz Shaikhyzada, Nazgul Abulkhanova, Akzhunis Altyn, Bakytkali Ibraimov, Dair Nurgaliyev, Dimitri Poddighe

**Affiliations:** 1 Program of Solid Oncology, Section of Pediatric Oncology, Clinical Academic Department of Pediatrics, National Research Center for Maternal and Child Health, University Medical Center (UMC), Astana 010000, Kazakhstan; 2 Section of Pathology, Clinical Academic Department of Laboratory Medicine, Republican Diagnostic Center, University Medical Center (UMC), Astana 010000, Kazakhstan; 3 Section of Pediatric Oncology, Clinical Academic Department of Pediatrics, University Medical Center (UMC), Astana 010000, Kazakhstan; 4 Associate Professor of Pediatrics, School of Medicine, Nazarbayev University, Kerei-Zhanibek Str. 5/1, Astana 010000, Kazakhstan; 5 Clinical Academic Department of Pediatrics, National Research Center for Maternal and Child Health, University Medical Center (UMC), Astana 010000, Kazakhstan

**Keywords:** extra-renal Wilms tumor, extra-renal nephroblastoma, spinal tumors, Pediatric Oncology, case-based review, developing countries

## Abstract

**Simple Summary:**

Wilms tumor (WT) is a rare form of cancer that typically affects children and is usually confined to the kidneys. Extra-renal Wilms tumor (ERWT) is even rarer and develops in other areas of the body, such as the retroperitoneum and inguinal regions, and occasionally at the level of the spinal cord. We present a case report of a 4-year-old boy diagnosed with spinal ERWT, who was also affected with a spinal dysraphism. Our case-based systematic review of pediatric ERWT showed that a multimodal therapeutic approach (including surgery, chemotherapy, and radiotherapy) is important, but an international standardization of the staging approach and therapeutic protocols is needed to define the best clinical management in these children: indeed, there is a lack of clinical studies focused on pediatric ERWT and international trials are needed to achieve these objectives. Our research emphasizes the importance of timely diagnosis and treatment and, possibly, standardized medical approach, in order to improve the outcome of these very rare pediatric malignancies, whose clinical management is even more problematic in developing countries.

**Abstract:**

Wilms Tumor (WT) is one of the most common renal tumors in the pediatric population. Occasionally, WT can primarily develop outside the kidneys (Extra-Renal Wilms Tumor, ERWT). Most pediatric ERWTs develop in the abdominal cavity and pelvis, whereas the occurrence of this tumor in other extra-renal sites represents a minor part of ERWT cases. In addition to describing a case of spinal ERWT (associated with spinal dysraphism) in a 4-year boy (to add a further clinical experience on this very rare pediatric tumor), we performed a case-based systematic literature review on pediatric ERWT. We retrieved 72 papers providing enough information on the diagnosis, treatment, and outcomes of 98 ERWT pediatric patients. Our research highlighted that a multimodal approach involving both chemotherapy and radiotherapy, after partial or complete tumor resection in most cases, was typically used, but there is no standardized therapeutic approach for this pediatric malignancy. However, this tumor may be potentially treated with a better success rate if the diagnostic confirmation is not delayed, the mass can be totally resected, and an appropriate and, possibly, tailored multimodal treatment can be promptly established. In this regard, an international agreement on a unique staging system for (pediatric) ERWT is definitely needed, as well as the development of international research, which may be able to gather several children diagnosed with ERWT and, possibly, lead to clinical trials which should also include developing countries.

## 1. Introduction

Wilms tumor (WT), or nephroblastoma, is one of the most common solid malignancies in children. It represents around 95% of renal tumors in the pediatric age, and, indeed, it arises almost exclusively from the kidneys [1]. However, the rare occurrence of extra-renal nephroblastoma (with no evidence of primary involvement of the kidneys) has been reported [2]. Extra-renal Wilms tumor (ERWT) was first described by Moyson et al. in 1961 and accounts for approximately 0.5 to 1% of WT diagnoses [3,4]. ERWT most often develops in the retroperitoneum and inguinal regions. However, it can arise from various sites, including the female genital organs (uterus, ovary, cervix), mediastinum, pelvis, adrenal glands, bladder, colon, prostate, scrotum, testis, lumbosacral region, paravertebral soft tissues, and spinal cord [2]. The most accredited pathogenic hypothesis is that ERWT can arise anywhere along the craniocaudal migration pathway of the primitive mesonephros and metanephros cells [2].

The clinical presentation of ERWT is unspecific and can vary according to the primary location and size of the mass. Useful diagnostic investigations are ultrasonography, computed tomography (CT), and magnetic resonance imaging (MRI); however, the radiological features of ERWT are also nonspecific: therefore, these imaging techniques alone cannot provide a final and safe diagnosis for this tumor; surgery and eventual histopathological examination are required to reliably distinguish ERWT from other malignancies, which may enter into the differential diagnosis (including primary intrarenal tumor with metastasis to the extra-renal site, teratomas with nephroblastoma components, other primitive mesenchymal tumors, etc.) [4,5].

We report a 4-year-old child diagnosed with ERWT arising in the spinal canal, an extremely rare location; moreover, this patient was also affected with spinal dysraphism. Additionally, we provide a systematic case-based review of pediatric ERWT cases described in the medical literature so far, in order to summarize and discuss the main diagnostic and therapeutic aspects and challenges.

## 2. Case Report

### 2.1. Clinical Presentation

A 4-year-old boy (without any previously known health problems) presented to the regional hospital because of intermittent limp and back/left leg pain for 2 months. According to his parents, such a problem appeared after falling on his back. No fever or other complaints were reported at that time. Family history was negative for any relevant diseases, including malignancies and congenital malformations.

In the regional hospital, the child underwent MRI of the spinal cord, which revealed a mass at the level of T12-S3 vertebrae; notably, this exam also revealed a spinal dysraphism (posterior spina bifida), which was not suspected or known before. However, any further medical assistance was refused until six months later, when they again brought their child to the same hospital after he had already developed paraplegia and other neurological dysfunctions (including urinary retention and intestinal constipation). A second MRI of the spinal cord revealed an intramedullary mass of the thoracic and lumbar spinal cord, with signs of extramedullary growth. Parents gave their consent for a biopsy of this mass, and the procedure was performed without any complications; unfortunately, mass excision was not possible.

After histopathological confirmation of malignancy, this patient was transferred to our referral national center for Pediatric Oncology, where the diagnostic work-up was completed, including brain, spinal cord, abdomen, and pelvis MRI with contrast medium, and chest CT, according to the recommendations of the Republic of Kazakhstan national medical protocols for patients diagnosed with any malignancy. The previously obtained histopathological material was also sent to our Pathology Department for further examination and analyses (see later), which supported the diagnosis of ERWT. Laboratory examinations showed no significant abnormalities except for mild anemia; however, bone marrow examination did not show any tumor infiltration. The levels of plasmatic α-fetoprotein, β-human chorionic gonadotropin, and neuron-specific enolase were within the normal range. A cerebrospinal fluid examination was not performed due to the contraindication represented by the extensive intraspinal tumor.

The main steps of the diagnostic timeline are reported in Table 1, along with the eventual clinical course and therapeutic management and follow-up.

### 2.2. Imaging

**Figure 1:**
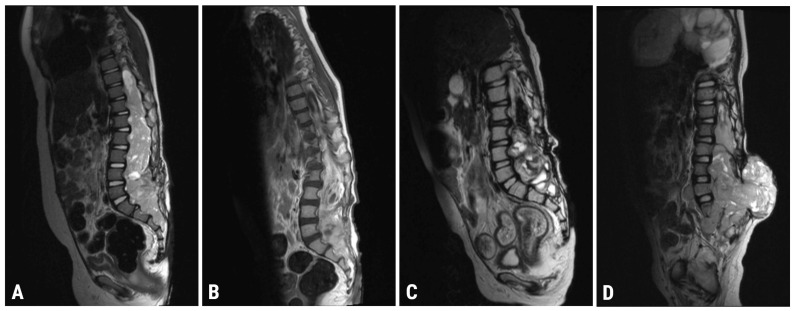
MRI images of the spinal cord in the sagittal projection, T2 weighted: (**A**) before starting chemotherapy; (**B**) before starting radiation therapy; (**C**) immediately after completion of chemotherapy and radiotherapy; (**D**) 6 months after completion of chemotherapy and radiotherapy.

**Figure 2:**
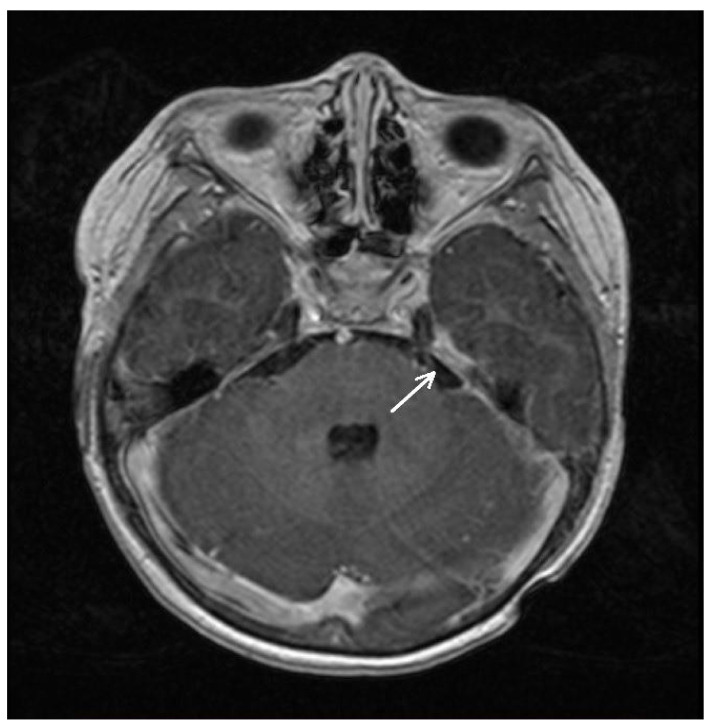
Axial MRI of the brain (T2 weighted). The arrow indicates the site of leptomeningeal metastasis of the tumor.

As mentioned, the MRI of the spinal cord was performed upon admission to our medical center: it revealed an irregularly shaped solid mass infiltrating the spinal canal at the T9-S4 level. The dimensions of the formation were up to 39 × 36 mm × 205 mm. At the T12-S1 level, the tumor spread paravertebrally on both sides (size: from 11 × 10 mm to 29 × 23 mm) along the nerve roots. Moreover, at the C1-C2 level, along the right-anterior surface of the spinal cord, an oval-shaped mass (with unclear margins and homogeneous structure; size: 7.4 × 6.4 mm) was also described (see Figure 1A). Finally, the brain MRI revealed an area (25 × 5.5 mm) of local accumulation of contrast medium in the pia mater meninx, consistent with leptomeningeal tumor metastasis. This finding was located in the medial part of the left temporal bone (Figure 2). Chest CT and MRI of the abdomen and pelvis were negative and, thus, the central nervous system as the only disease site.

The main radiological findings during the clinical course and follow-up are also summarized in Table 1.

### 2.3. Histopathological Examination

**Figure 3:**
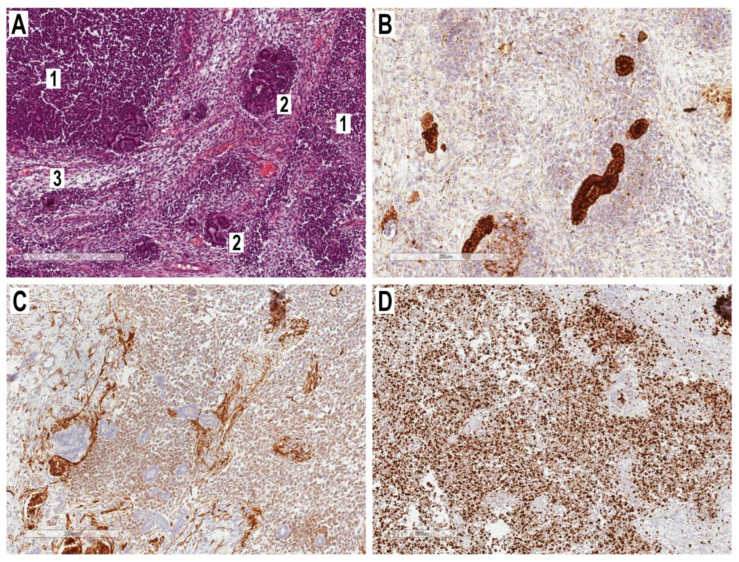
Main histopathological findings: (**A**) 1—blastema, 2—epithelial, 3—stromal components, hematoxylin-eosin stain (×300); (**B**) Pan-cytokeratin (AE1/AE3)—positive reaction of the epithelial component, immunohistochemical staining (×200); (**C**) WT-1—weakly positive reaction of the blastema component, immunohistochemical staining (×200); (**D**) Ki-67—the high proliferative activity of tumor cells, immunohistochemical staining (×300).

The histopathological examination revealed a tumor mass represented by blastemal, epithelial, and stromal components. The blastemal component was characterized by foci of medium-sized cells having round and oval hyperchromic nuclei and poor cytoplasm; multiple mitoses were noted. The epithelial component was characterized by formations resembling renal tubules lined with cuboidal epithelium with rounded nuclei and light cytoplasm, and primitive glomerular structures. The stromal component was represented by patches of fibrous tissue (Figure 3).

The immunohistochemistry of tumor cells was WT1—diffuse positive, Pan-cytokeratin epithelial cells—focally positive, CD99—positive, NSE—stroma positive, Desmin—negative, and S100—negative. In addition, the proliferative activity of tumor cells (Ki-67) reached 90%.

Thus, the morphological and immunohistochemical characteristics were consistent with nephroblastoma and, thus, spinal ERWT (due to the absence of primary kidney location, according to the radiological work-up discussed in the previous section) was finally diagnosed.

**Table 1:** Clinical, diagnostic, and therapeutic chronological timeline.

January 2020	Clinical onset (intermittent limp and back/left leg pain)
April 2020	Spinal MRI (mass in the spinal canal at the T12-S3 level)
11 November 2020	Spinal MRI (confirmation of increased intramedullary mass, with signs of extramedullary growth)
18 December 2020	Mass biopsy (diagnosis: extra-renal nephroblastoma of the spinal cord)
8 January 2021	Spinal MRI (T9-S4 mass: 39 × 36 × 205 mm; & C1-C2 mass: 7.4 × 6.2 mm; Figure 1A) Brain MRI (leptomeningeal metastasis of the left temporal lobe: 25 × 5.5 mm)
9 January 2021	Histopathological examination (ERWT confirmation)
14 January 2021	Post-operative adjuvant chemotherapy (1st cycle)
2 March 2021	Spinal MRI (T9-S4 mass: 39 × 33 × 193 mm; & C1-C2 mass: 6 × 3 mm) Brain MRI (unchanged leptomeningeal metastasis of the left temporal lobe: 25 × 5.5 mm in size)
12 April 2021	Completion of post-operative adjuvant chemotherapy (5th cycle)
4 May 2021	Spinal MRI (T9-S4 mass: 38 × 32 × 193 mm; & C1-C2 mass: 7 × 3 mm; Figure 1B) Brain MRI (unchanged leptomeningeal metastasis of the left temporal lobe: 25 × 5.5 mm in size)
13 May 2021	Post-operative adjuvant radiotherapy (1st session)
7 July 2021	Completion of post-operative adjuvant radiotherapy (34th session)
30 July 2021	Spinal MRI (T9-S4 mass: 37 × 32 × 190 mm; C1-C2 mass: 7 × 3 mm) Brain MRI (leptomeningeal metastasis of the left temporal lobe: 22 × 4 mm in size)
16 October 2021	Completion of post-operative adjuvant chemotherapy (9th cycle)
22 October 2021	Spinal MRI (T9-S4: 36 × 32 × 190 mm; & C1-C2 mass: 7 × 3 mm; Figure 1C) Brain MRI (leptomeningeal metastasis of the left temporal lobe: 20 × 4 mm)
13 December 2021	PET-CT (high metabolic activity: spinal canal) (weak metabolic activity: neck, left axillary, and right inguinal lymph nodes)
14 December 2021	Second tumor biopsy
23 December 2021	Histopathological re-examination (confirmed diagnosis of ERWT)
6 April 2022	Spinal MRI (T11-S2 mass: 76 × 75 × 195 mm; & C1-C2 mass: 7 × 5 mm; Figure 1D) Brain MRI (leptomeningeal metastasis of the left temporal lobe: 17 × 6 mm in size)
8 April 2022	Chest CT (multiple bilateral lung metastases, left-sided pneumothorax)
9 April 2022	Palliative chemotherapy course
17 April 2022	Death

MRI, magnetic resonance imaging; CT, computed tomography; PET-CT, positron emission tomography-computed tomography; ERWT, extra-renal Wilms tumor.

### 2.4. Medical Management and Clinical Course

After the final ERWT diagnostic confirmation, chemotherapy was started according to the protocol of the International Society for Pediatric Oncology (SIOP WT 2001), stage III, in the high-risk group (due to the presence of metastases). Nine courses of chemotherapy were performed, consisting of etoposide (150 mg/m^2^), carboplatin (200 mg/m^2^), cyclophosphamide (450 mg/m^2^), and doxorubicin (50 mg/m^2^). Additionally, the patient underwent radiation therapy, receiving 25.5 Gy in 17 fractions on the craniospinal axis and an additional sequential booster dose of 25.5 Gy in 17 fractions on the main tumor site. The overview of the clinical course and therapeutic management is summarized in Table 1.

After 11 months from the beginning of the multimodal therapy (in December 2021), due to the lack of significant response, a second and more extensive tumor biopsy was performed. Histopathological and immunohistochemical examination of tumor cells once again confirmed the diagnosis of ERWT. Positron emission tomography-computed tomography (PET-CT), performed before this second biopsy, also confirmed a metabolically active mass in the spinal canal; notably, cervical, left axillary, and right inguinal lymph nodes uptake with low metabolic activity were also detected.

After this diagnostic reassessment, the patient was discharged from the hospital due to a break between chemotherapy courses, but he returned to our medical center only four months later. The spinal MRI described an increased size of the mass, as shown in Figure 1D. The brain MRI still confirmed the presence of the known leptomeningeal metastatic focus. The chest CT also showed a mediastinal mass and multiple lung metastases.

This unfortunate patient started a palliative course with ICE chemotherapy (ifosfamide: 2000 mg/m^2^; carboplatin: 500 mg/m^2^; and etoposide: 100 mg/m^2^), which was interrupted due to the rapid deterioration of the clinical condition. The patient died in the intensive care unit around two years after his initial ERWT diagnosis.

## 3. Case-Based Review

### 3.1. Systematic Literature Search

A systematic case-based review was done through an extensive literature review in Pubmed and Scopus databases. The search used the following terms: “extra-renal Wilms’ tumour” OR “extra-renal nephroblastoma”. All pediatric case reports and series describing at least one pediatric patient diagnosed with ERWT were extracted. If any, original articles describing clinical studies, including pediatric ERWT patients, were extracted and considered in the discussion. Letters, editorials, review articles, and, in general, all articles which did not provide a minimal clinical description of ERWT pediatric patients were excluded. Only English-language articles were included. The search period ran from 1961 (when the ERWT was first described in a pediatric patient) until 31 December 2022.

**Figure 4:**
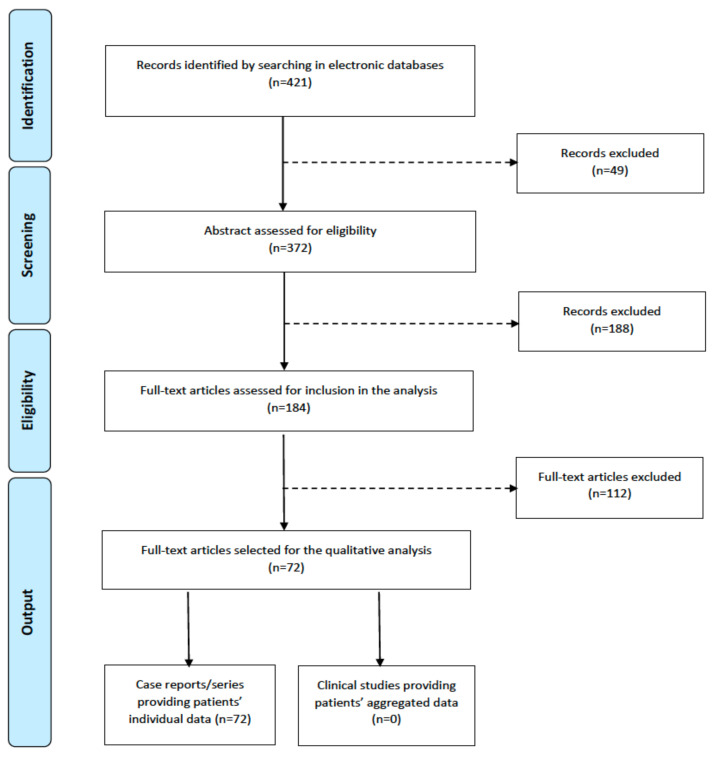
PRISMA flowchart showing the pediatric ERWT systematic literature review.

A total of 421 items were retrieved from the medical literature in the electronic database; after excluding duplicated records and inappropriate manuscripts (review articles, abstracts, conference papers, and non-English publications), and after screening the article abstracts, 237 titles were discarded. Thus, 184 titles were considered for eligibility: a total of 72 full-text accessible papers were selected since these included at least one pediatric EWTW and provided minimal clinical, diagnostic, therapeutic, and follow-up information. The PRISMA flowchart describing all the stages of this systematic literature search is shown in Figure 4.

### 3.2. Data Extraction

After a critical assessment and selection of the articles according to the PRISMA guidelines, data extraction was done by one investigator and was checked by a second investigator following these main inclusion criteria: any case report/series articles including pediatric patients diagnosed with ERWT, which could provide sufficient information on clinical, diagnostic, therapeutic and follow-up aspects, according to the objectives of the data extraction, as described below.

The following information was extracted: first author’s last name, publication year, patient’s gender and age, ERWT location, time of diagnosis, tumor stage, treatment, relapse, metastasis, follow-up, and outcome.

## 4. Results

The final output of this systematic case-based literature review consisted of 72 papers, including case reports (*n* = 59) and small case series (*n* = 13). Notably, no clinical studies investigating or focused on ERWT pediatric patients (including data in aggregated form) were retrieved.

The main patient’s data extracted from case reports and series are schematically summarized in Table 2. In summary, the output consisted of 98 ERWT pediatric patients. The tumor site distribution was as follows: retroperitoneum (*n* = 51), inguinal region (*n* = 13; in detail: inguinal canal, *n* = 9; unspecified location, *n* = 4), female genital tract (*n* = 8; in detail: uterus, *n* = 4; ovary, *n* = 3; cervix, *n* = 1), (para-)vertebral/spinal regions (*n* = 12; in detail: lumbar, *n* = 6; thoracolumbar, *n* = 1; lumbosacral, *n* = 1; thoracolumbosacral, *n* = 2; sacrocoggigeal region, *n* = 1; unspecified paravertebral region, *n* = 1), pelvis (unspecified site, *n* = 5), bladder (*n* = 3), scrotum (*n* = 3), chest wall (*n* = 1), colon (*n* = 1, in a patient affected with sigmoidal tract duplication), mesentery (*n* = 1).

Overall, the gender ratio of this pooled population was 1.06:1 (female, *n* = 50; males, *n* = 47; gender information was not available for one pediatric patient). Age information was available for 92 patients (out of 98): overall, the age of these pediatric ERWT patients at diagnosis was 4.02 ± 3.83 years (mean ± standard deviation).

Clear staging information for pediatric ERWT was available for only 42 patients (out of 98). Among these 42 cases with ERWT staging information, 28 were staged according to the National Wilms Tumor Study (NWTS) for intrarenal Wilms tumors. In two cases, the United Kingdom Children’s Cancer Study Group protocol was used for staging. In five cases, the staging system, according to the International Society of Pediatric Oncology (SIOP), was used. In two cases, staging according to the TNM staging system was applied. In the remaining five cases, the staging system was not clearly specified. According to the NWTS (*n* = 28), three cases were diagnosed as stage I, 11 cases as stage II, 12 cases as stage III, and 2 cases as stage IV. According to the SIOP staging system (*n* = 5), one case was at stage I, three cases at stage III, and one case at stage IV. Additionally, according to the United Kingdom Children’s Cancer Study Group (*n* = 2), both patients were categorized as stage III. Lastly, based on the TNM (tumor, node, metastasis) system (*n* = 2), these patients were classified as stage I and stage III, respectively.

Information about the therapeutic management was available for the majority of ERWT children. Most patients were treated using a multimodal approach (*n* = 84; in detail: surgery + chemotherapy, *n* = 41; surgery + radiation therapy, *n* = 2; surgery + chemoradiotherapy, *n* = 41). A minority of patients (*n* = 12) underwent surgery only. Overall, almost all patients (*n* = 93) underwent partial or complete resection of the tumor, except three children who received only tumor biopsy; moreover, no clear information is given about two patients.

In terms of clinical course, 16 patients developed recurrence or metastases (clear information is available for 81 patients out of 98). Among these relapsed patients, local recurrence was described in five cases, whereas seven patients developed metastases distant from the primary tumor site (such as lungs, liver, pulmonary and mediastinal lymph nodes, cerebellum, and peritoneum). In four patients, both local relapse and metastases were concomitantly diagnosed (as occurred in our case report).

The mean follow-up was 2.49 *±* 2.05 years, based on data from 78 patients; indeed, unfortunately, no follow-up period is available for the remaining 20 ERWT cases. This period varied between one month and 10.8 years. As regards the outcome analysis, 77 patients were alive at the end of the follow-up, and death was reported in five cases. Finally, there is information on the time elapsed between symptoms onset and ERWT diagnosis for 47 patients: the estimated median time was approximately 12–13 weeks, but unfortunately, some patients needed several months to be diagnosed (6–12 months, *n* = 7; >12 months: *n* = 2).

Since our case report described a child affected with ERWT located in the spinal cord, we performed a subanalysis of these patients, as shown in Table 3. Here, we focused on and highlighted peculiar clinical characteristics and specific therapeutical aspects.

## 5. Discussion

Pediatric ERWT is a rare malignancy; indeed, all available articles are case reports or small case series, as shown by our systematic literature research. The primary site is extremely variable since this malignancy can develop anywhere along the cranio-caudal migration pathway of primitive mesonephros and metanephros cells [2]. Retroperitoneum, inguinal region/scrotum (in males), and the female genital organs are the most prevalent sites, which account for at least 70% of the ERWT cases which have been included in this systematic review. Accordingly, the clinical manifestations of pediatric ERWT are highly variable, depending on the primary site and extension of the mass, in addition to its stage. Unless ERWT is pre-clinically detected as an asymptomatic but palpable abdominal mass, it may manifest with nonspecific symptoms (e.g., abdominal pain/discomfort, weight loss, hematuria, vaginal bleedings or discharge, lymphadenopathy, etc.) or, like in our case, neurological manifestations, if there is compression of the spinal cord and/or infiltration of nervous structures.

Moreover, we also highlighted that there is no standardized treatment protocol for ERWT in children; however, the therapeutic approach is multimodal, although it is firstly based on the surgery, which is also essential for the diagnostic confirmation [2]. According to our analysis, almost all pediatric patients (96.9%) underwent partial or total surgical removal of ERWT; after surgery, most of them (84.9%) received chemotherapy, and among these, around a half (51.9%) also underwent concomitant radiotherapy. Whereas the indication and modality of radiotherapy were determined by the primary tumor site, stage, histological variant, presence of metastases, and tumor recurrence, the chemotherapy regimens were mainly based on a combination of vincristine and actinomycin D.

Despite such a therapeutic heterogeneity, patient’s death was reported in only five cases (6.1%) at the end of 2.5-year median follow-up; therefore, the lack of a longer follow-up does not allow us to know if there were mid/long-term relapsed cases and, thus, the actual mortality rate. According to two international multidisciplinary cooperative consortia—the Children’s Oncology Group (COG) Renal Tumour Committee, previously known as the National Wilms Tumour Study Group (NWTSG), and the International Society of Paediatric Oncology (SIOP) Renal Tumour Study Group (RTSG), despite different treatment approaches, the overall survival of WT patients is approximately 90% [76]. In patients with stage IV anaplastic WT and/or blastemal type WT, outcomes are significantly worse: overall survival <50% despite very intensive therapy [76,77,78]. Such an apparently “satisfactory” survival rate may be due to a multitude of factors, which is not possible to clearly analyze and understand without controlled clinical studies. Performing tumor excision with adequate lymph node sampling (though it is universally done well with renal WT) [76] may positively impact therapeutic choices and survival. This approach is recommended by both SIOP and COG; however, the timing of surgery differs between the SIOP and COG recommendations and underpins the differences in risk stratification [79,80].

The knowledge of two effective drugs (vincristine and actinomycin D) in WT may have further improved the survival of ERWT children. This combination of drugs was developed by the SIOP and the NWTS in the 1970s and 1980s, and was shown to be highly effective in treating renal WT. As a result, this combination has been widely used in treating nearly two-thirds of children diagnosed with this disease [81,82], including extra-renal forms. Moreover, the addition of doxorubicin to this chemotherapy regimen has been found to further benefit some WT patients, especially those with metastatic disease and high-risk histological subtypes [83,84,85]. Unfortunately, this information is variably provided in the articles included in our systematic literature review, and we cannot have a reliable (even if approximative) estimation of the effective use of these three drugs in our pool of ERWT children. Moreover, the good radiosensitivity of nephroblastoma neoplastic cells may also have contributed to some extent [86]. The NWTS study demonstrated the efficacy of radiotherapy for renal WT, particularly in preventing abdominal recurrence due to potential tumor spillage after surgery. Patients receiving two- or three-component chemotherapy without radiation therapy had a significantly higher frequency of abdominal recurrences. In contrast, a dose of 10 Gy radiotherapy resulted in a reduction of tumor recurrence after surgical excision and a 20 Gy dose was even more effective [86].

Therefore, even though no general conclusion can be made in terms of optimal therapeutic management (especially as regards specific chemotherapy and/or radiotherapy regimens), the recommendation for a total surgical resection (whenever this is possible) is the therapeutic mainstay of pediatric ERWT inside a multimodal and personalized therapeutic plan, which should take into consideration the tumor site, histological details, and staging. As regards the chemotherapy, vincristine, actinomycin D, and doxorubicin were among the most used drugs to treat pediatric ERWT, as also discussed by Liang et al., who reported the largest monocentric case series (five patients) so far [72]; however, many case reports do not describe the therapeutic approach in detail, especially as regards the chemotherapy regimens: therefore, it is not possible to give specific recommendations on this matter without any controlled and appropriately designed clinical studies on pediatric ERWT. The application of radiotherapy is even less standardized: it is usually reserved for patients with unresectable tumors, gross residues, recurrence, or metastasis, as highlighted by several authors [2,44,64].

As specifically regards the staging, our literature research definitely highlighted the lack of a uniform approach. The use of different staging systems (COG, SIOP, UKCCSG/CCLG, and TNM) was observed. The staging system for renal WT was developed by the National Wilms Tumor Study Group (NWTS) and updated by the Renal Tumors Committee of the Children’s Oncology Group (COG). SIOP is another European Group that has provided a different WT staging system since 1971: this differed from COG regarding the concept of giving preoperative chemotherapy to all patients > 6 months of age [87]. Neoadjuvant chemotherapy allows assessment of in vivo histological response to treatment (basically, a completely necrotic tumor indicates high responsiveness while a predominance of remaining blastemal cells is a marker of chemotherapy resistance), which may be used to guide therapeutic stratification after nephrectomy [76]. CCLG also adopted the SIOP WT staging system because the UK-CCLG-SIOP 2001 Study (2001–2011) was a part of the SIOP-WT-2001 Study, which registered patients with renal tumors from all CCLG centers [88]. Finally, the TNM classification was also used in some pediatric ERWT reports; this is a staging system used in general for solid tumors, which is based upon local tumor spread, involvement of regional lymph nodes, and presence of distant metastasis [89]. Despite the highly heterogeneous approach for pediatric ERWT staging, in the vast majority of cases, the first line of treatment was the surgical resection of the mass. Following surgery and histological confirmation, staging the tumor according to the COG criteria at the time of the surgery could be more applicable in this pathological context, since the surgical approach resulted in being the first step, whenever possible. Conversely, the SIOP staging is based on the administration of preoperative chemotherapy to all patients with WT, which was not the main and initial approach in the context of pediatric ERWT, according to our literature review. Moreover, in general, the definition of stage I could be revised to define the localized tumor that can be entirely resected with clear microscopic margins, thus without any residual disease or rupture of the tumor during surgery. In summary, an international consensus for a uniform staging strategy in pediatric ERWT patients is needed, which would be the preliminary step toward implementing standardized treatment protocols.

As an additional completion of our analysis, we also focused on pediatric ERWT arising from the spinal cord region, starting from our direct experience with our patient. As summarized in Table 3, this subgroup of pediatric ERWT patients was relatively younger than all other ERWT children (respectively, 2.25 ± 1.54 years vs. 4.02 ± 3.83), and, interestingly, all these children were female, except our case. A spinal cord malformation was present in all cases, which was also associated with an external malformation present in four patients (out of six). Total excision was possible in 4 cases, and all these patients received variable chemotherapy regimens; moreover, most patients also underwent radiotherapy. At the end of the follow-up (1.38 ± 0.94 years), 4 patients were still alive.

In perspective, clinical studies are crucial for developing new treatments for rare tumors, including ERWT. Of course, conducting clinical trials for rare tumors is challenging due to the small patients’ population and, thus, the related logistic issues to include study participants from different areas of the world, along with the hesitancy from the pharmaceutical industry to specifically invest in “small markets” clinical research [90]. Moreover, the complexity of performing these clinical trials is even greater if we consider the diagnostic and/or therapeutic limitations in resource-limited settings (including Kazakhstan), where the (pediatric) population is often more numerous than in most developed countries and, thus, the potential contribution to clinical trials in terms of potential number of study participants may be remarkable [91,92]. Indeed, in these countries, several diagnostic barriers can impair clinical research in general and, in detail, the development of controlled clinical trials, as we also have discussed recently, as regards several pediatric fields, not limited to oncology only [93,94,95]. However, these studies are essential to improve our understanding of the disease mechanisms and, thus, developing effective and tailored treatments.

## 6. Conclusions

Pediatric ERWT is a rare form of cancer that must be diagnosed and treated with a multimodal approach. Currently, there is no standardized therapeutic approach for this pediatric malignancy, as is highlighted by the present article. However, this tumor could be potentially treated with a good success rate if the certain diagnosis is not delayed, the mass can be totally resected, and an appropriate and, possibly, tailored multimodal treatment can be promptly established. In this regard, an international agreement on a unique staging system for (pediatric) ERWT is definitely needed, as well as the development of international research, which may be able to gather a number of children diagnosed with ERWT and lead to clinical trials, which should also include developing countries.

## Figures and Tables

**Table 2:** Summary of the pediatric ERWT patients included in this case-based systematic review.

№	Author	Year	Sex	Age (yrs.)	ERWT Origin Site	Diagnostic Time (wks.)	Stage	Treatment	Relapse/Metastasis	Follow-Up (yrs.)	Outcome
1	Bhajkar et al. [6]	1964	M	2	Retro- peritoneum	26	n/a	Surgery + RAD	None	0.8	Alive
2	Edelstein et al. [7]	1965	M	3	Retro- peritoneum	13	n/a	Surgery + chemo + RAD	None	0.7	Alive
3	Thompson et al. [8]	1973	F	4.5	Inguinal region	1	n/a	Surgery + RAD	Local	2	Alive
4	Thompson et al. [8]	1973	M	3	Inguinal canal	n/a	n/a	Surgery + chemo + RAD	Local + Right lung	0.5	Death
5	Akhtar et al. [9]	1977	M	0.2	Inguinal canal	5	n/a	Surgery	None	1.5	Alive
6	Madanat et al. [10]	1978	F	9	Chest wall	n/a	III (NWTS)	Surgery * + chemo + RAD	None	2.7	Alive
7	Madanat et al. [10]	1978	M	0.3	Inguinal canal	6	I (NWTS)	Surgery + chemo	None	1.8	Alive
8	McCauley et al. [11]	1979	F	4.5	Retro- peritoneum	0.3	III (NWTS)	Surgery + chemo + RAD	None	4	Alive
9	Johnson et al. [12]	1980	F	1.5	Retro- peritoneum	0.1	I	Surgery + chemo	None	1	Alive
10	Fried et al. [13]	1980	M	3.5	Retro- peritoneum	0.1	n/a	Surgery + chemo	n/a	n/a	Alive
11	Orlowski et al. [14]	1980	M	3.5	Scrotum	n/a	n/a	Surgery + chemo + RAD	Left lung	1.5	Alive
12	Taylor et al. [15]	1980	M	0.5	Scrotum	n/a	n/a	Surgery + chemo + RAD	None	0.5	Alive
13	Bittencourt et al. [16]	1981	F	14	Uterus	52	n/a	Surgery + chemo + RAD	None	5.7	Alive
14	Adam et al. [17]	1983	M	10	Retro- peritoneum	n/a	n/a	Surgery	None	0.1	Alive
15	Meng et al. [18]	1983	M	3	Retro- peritoneum	9	n/a	Surgery	n/a	n/a	n/a
16	Lüchtrath et al. [19]	1984	F	1.2	Inguinal region	48	n/a	Surgery + chemo	None	3	Alive
17	Fernbach et al. [20]	1984	F	2	Spinal cord (L1)	n/a	n/a	Surgery + chemo + RAD	None	1	Alive
18	Lai et al. [21]	1988	F	5	Inguinal region	n/a	n/a	Surgery + chemo	Local	1.6	Alive
19	Narasimharao et al. [22]	1989	M	2	Retro- peritoneum	13	n/a	Surgery + chemo	None	1	Alive
20	Fernandes et al. [23]	1989	M	6	Retro- peritoneum	0.7	III (NWTS)	Surgery + chemo + RAD	None	7	Alive
21	Fernandes et al. [23]	1989	F	2	Retro- peritoneum	n/a	II (NWTS)	Surgery + chemo	None	5	Alive
22	Fernandes et al. [23]	1989	F	2	Retro- peritoneum	n/a	II (NWTS)	Surgery + chemo	None	1	Alive
23	Wakely et al. [24]	1989	F	0.8	Uterus	n/a	n/a	Surgery + chemo	None	2	Alive
24	Wakely et al. [24]	1989	F	1.8	Retro- peritoneum	n/a	n/a	Surgery + chemo + RAD	None	6	Alive
25	Wakely et al. [24]	1989	F	4	Retro- peritoneum	3	n/a	Surgery + chemo	None	5	Alive
26	Wakely et al. [24]	1989	M	4	Retro- peritoneum	n/a	n/a	Surgery + chemo + RAD	None	6	Alive
27	Broecker et al. [25]	1989	F	0.8	Pelvis	n/a	II (NWTS)	Surgery + chemo	None	1	Alive
28	Broecker et al. [25]	1989	F	1.8	Retro- peritoneum	n/a	n/a	Surgery + chemo + RAD	None	7	Alive
29	Broecker et al. [25]	1989	F	1.8	Retro- peritoneum	n/a	II (NWTS)	Surgery + chemo	Lung node	1.8	n/a
30	Strand et al. [26]	1990	M	0.9	Inguinal canal	48	n/a	Surgery + chemo	n/a	n/a	n/a
31	Mirkin et al. [27]	1990	F	2	Spinal cord (T12-L4)	n/a	n/a	Surgery + chemo + RAD	Cerebellum	1.7	Alive
32	Sarode et al. [28]	1992	M	2	Retro- peritoneum	9	n/a	Surgery + chemo	n/a	n/a	n/a
33	Andrews et al. [29]	1992	F	n/a	Sacrococcygeal region	n/a	II (NWTS)	Surgery + chemo	None	1.3	Alive
34	Andrews et al. [29]	1992	M	n/a	Retro- peritoneum	n/a	II (NWTS)	Surgery + chemo	None	0.6	Alive
35	Andrews et al. [29]	1992	F	n/a	Lumbar region	n/a	II (NWTS)	Surgery + chemo	None	6.2	Alive
36	Andrews et al. [29]	1992	M	n/a	Retro- peritoneum	n/a	IV (NWTS)	Surgery + chemo + RAD	Lungs	2	Death
37	Andrews et al. [29]	1992	F	n/a	Retro- peritoneum	n/a	I (NWTS)	Surgery + chemo	None	2.8	Alive
38	Andrews et al. [29]	1992	F	n/a	Pelvis	n/a	II (NWTS)	Surgery + chemo	Lungs	4	Alive
39	Suzuki et al. [30]	1993	M	2	Retro- peritoneum	n/a	n/a	Surgery	n/a	n/a	n/a
40	Rasheed et al. [31]	1993	M	3	Retro- peritoneum	3	III (UKCCSG)	Surgery + chemo + RAD	None	7	Alive
41	Rasheed et al. [31]	1993	F	4	Retro- peritoneum	1.4	III (UKCCSG)	Surgery + chemo + RAD	None	1.7	Alive
42	Mount et al. [32]	1996	F	5	Retro- peritoneum	n/a	n/a	Surgery + chemo	None	2	Alive
43	Arkovitz et al. [33]	1996	M	3.5	Inguinal canal	n/a	III (NWTS)	Surgery + chemo + RAD	None	2	Alive
44	Kapur et al. [34]	1998	M	1.5	Retro- peritoneum	n/a	I (TNM)	Surgery + chemo	None	0.6	Alive
45	Kapur et al. [34]	1998	M	2	Retro- peritoneum	2	III (TNM)	Surgery + chemo + RAD	None	3	Alive
46	Benatar et al. [35]	1998	F	11	Uterus	n/a	n/a	Surgery	Local	0.6	n/a
47	Babin et al. [36]	2000	F	13	Uterus	9	n/a	Surgery + chemo + RAD	Local	5	Alive
48	Govender et al. [37]	2000	F	4	Spinal cord (T10-Sx)	13	n/a	Surgery * + chemo + RAD	n/a	n/a	n/a
49	Arda et al. [38]	2001	F	5	Retro- peritoneum	n/a	III	Surgery + chemo + RAD	None	3	Alive
50	Oner et al. [39]	2002	F	3.5	Ovary	n/a	n/a	Surgery + chemo	None	0.6	Alive
51	Deshpande et al. [40]	2002	M	1	Lumbar region (L2-L4)	9	n/a	Surgery + chemo + RAD	n/a	n/a	Alive
52	Yunus et al. [41]	2003	M	<0.1	Retro- peritoneum	0.7	n/a	Surgery + chemo	None	1.8	Alive
53	Apoznański et al. [42]	2005	M	17	Retro- peritoneum	n/a	III (SIOP)	Surgery + chemo + RAD	None	1	Alive
54	Sharma et al. [43]	2005	F	1.5	Spinal cord (L2-5)	n/a	n/a	Surgery + chemo	n/a	n/a	Alive
55	Sastri et al. [44]	2006	M	2	Paravertebral region	26	n/a	Surgery + chemo + RAD	None	5	Alive
56	Sastri et al. [44]	2006	M	0.8	Lumbar region	0.7	n/a	Surgery + chemo + RAD	None	4	Alive
57	Sastri et al. [44]	2006	F	15	Retro- peritoneum	9	n/a	Surgery + chemo + RAD	None	5	Alive
58	Houben et al. [45]	2007	M	3.7	Retro- peritoneum	n/a	IV (NWTS)	Surgery + chemo	None	4	Alive
59	Houben et al. [45]	2007	M	2.8	Retro- peritoneum	n/a	I (NWTS)	Surgery + chemo	None	1	Alive
60	Ramachandra et al. [46]	2007	M	4	Retro- peritoneum	8	III (NWTS)	Surgery + chemo + RAD	None	1	Alive
61	Ramachandra et al. [46]	2007	F	3	Retro- peritoneum	n/a	II (NWTS)	Surgery + chemo + RAD	None	1.3	Alive
62	Leblebici et al. [47]	2009	F	16	Uterus	26	n/a	Surgery + chemo	n/a	n/a	Death
63	Jiaet al. [48]	2009	F	3	Retro- peritoneum	1.4	n/a	Surgery	n/a	0.3	n/a
64	Ngan et al. [49]	2009	F	6	Retro- peritoneum (juxtarenal)	0.7	I	Surgery	None	1	Alive
65	Cooke et al. [5]	2009	M	1.2	Inguinal canal	n/a	n/a	Surgery	None	3	Alive
66	Imran et al. [50]	2010	F	7	Retro- peritoneum	n/a	n/a	Surgery + chemo + RAD	None	n/a	Alive
67	Taguchi et al. [4]	2010	F	2.8	Retro- peritoneum	n/a	n/a	Surgery + chemo	None	2	Alive
68	Teerthanath [51]	2011	F	6	Retro- peritoneum	26	n/a	Surgery + chemo	None	4	Alive
69	Jeong et al. [52]	2011	M	9	Inguinal canal	1.4	n/a	Surgery + chemo	Lungs, mediastinal lymph nodes	n/a	n/a
70	Yamamoto et al. [53]	2012	M	0.6	Scrotum	n/a	n/a	Surgery	None	3	Alive
71	Armanda et al. [54]	2012	F	0.1	Lumbosacral region	1.4	I (SIOP)	Surgery + chemo	None	2	Alive
72	Li et al. [55]	2012	F	1.8	Pelvis	2	III (NWTS)	Surgery + chemo + RAD	None	3	Alive
73	Gordetsky et al. [56]	2012	M	17	Retro- peritoneum (juxtarenal)	9	II	Surgery + chemo + RAD	n/a	n/a	n/a
74	Marwah et al. [57]	2012	F	1.2	Ovary	n/a	n/a	Surgery + chemo	n/a	n/a	n/a
75	Hiradfar et al. [58]	2012	F	9	Inguinal region	n/a	n/a	Surgery	n/a	n/a	n/a
76	Rojas et al. [59]	2013	M	2	Retro- peritoneum	n/a	I/II	Surgery + chemo	n/a	n/a	n/a
77	Morandi et al. [60]	2013	M	3	Pelvis	n/a	n/a	Surgery + chemo	None	2	Alive
78	Goel et al. [61]	2014	n/a	5	Retro- peritoneum	9	n/a	Surgery + chemo + RAD	None	2	Alive
79	Kumar et al. [62]	2015	F	7	Retro- peritoneum	1	n/a	Surgery	None	0.8	Alive
80	Thakkar et al. [63]	2015	F	5	Retro- peritoneum	3	III (NWTS)	Surgery + chemo + RAD	None	n/a	Alive
81	Park [64]	2016	F	4	Retro- peritoneum	n/a	n/a	Surgery + chemo + RAD	Lungs, peritoneum	4	Alive
82	Wabada et al. [65]	2017	M	2	Retro- peritoneum	13	III (SIOP)	Surgery + chemo	None	0.3	Alive
83	Itoshima et al. [66]	2017	M	4	Retro- peritoneum	n/a	III (NWTS)	Surgery + chemo + RAD	None	3	Alive
84	Igbaseimokumo et al. [67]	2017	F	<0.1	Spinal cord (L5)	13	n/a	Surgery + chemo	None	2.5	Alive
85	Tang et al. [68]	2018	M	2	Retro- peritoneum	n/a	n/a	n/a	n/a	n/a	n/a
86	Tang et al. [68]	2018	F	2	Mesentery	n/a	n/a	n/a	n/a	n/a	n/a
87	Sindhu et al. [69]	2019	M	6	Bladder	65	III (SIOP)	Surgery + chemo + RAD	None	n/a	Alive
88	Groth et al. [70]	2019	M	0.7	Inguinal canal	n/a	III (NWTS)	Surgery + chemo + RAD	Local	1.3	Alive
89	Ismy et al. [71]	2019	M	1	Bladder	13	n/a	Surgery	n/a	n/a	n/a
90	Liang et al. [72]	2020	M	5	Retro- peritoneum	n/a	III (NWTS)	Surgery + chemo	Local + Lungs + Liver	1	Death
91	Liang et al. [72]	2020	F	3.4	Retro- peritoneum	n/a	III (NWTS)	Surgery + chemo + RAD	None	10.8	Alive
92	Liang et al. [72]	2020	F	3.4	Sigmoid colon	4	II (NWTS)	Surgery + chemo	None	3.3	Alive
93	Liang et al. [72]	2020	M	9.8	Retro- peritoneum	n/a	III (NWTS)	Surgery + chemo + RAD	Local + Lungs	1.8	Alive
94	Liang et al. [72]	2020	M	2.8	Inguinal canal	n/a	II (NWTS)	Surgery + chemo	None	1.5	Alive
95	Parkhi et al. [73]	2022	F	4	Bladder	4	n/a	Surgery + chemo	None	0.8	Alive
96	Qu et al. [74]	2022	M	0.5	Pelvis	4	n/a	Surgery + chemo	None	0.3	Alive
97	Albiroty et al. [75]	2022	F	2	Ovary	9	n/a	Surgery + chemo + RAD	None	1	Alive
98	Our case	2022	M	4	Spinal cord (T9-S4)	48	IV (SIOP)	Surgery * + chemo + RAD	Local + Lungs	0.3	Death

Abbreviations: F, female; M, male; yrs., years; wks., weeks; n/a, information not available; NWTS, National Wilms Tumor Study; UKCCSG, United Kingdom Children’s Cancer Study Group; TNM, TNM classification system of malignant tumors (Tumor, Node, Metastasis); SIOP, International Society of Paediatric Oncology; chemo, chemotherapy; RAD, radiotherapy. * In these 3 case reports, the authors declared that only biopsy was performed without partial or total tumor resection.

**Table 3:** Literature review of the pediatric cases of ERWT of the spinal cord, in addition to the present case.

№	Article	Sex	Age (yrs.)	Site	Spinal Malformation	External Malformation	Surgery	Chemo-Therapy (Main Drugs)	Radio- Therapy (Regimen)	Recurrence/ Metastasis	Follow-Up (yrs.)	Outcome
1	Fernbach et al., 1984 [26]	F	2	L1	Diastemato-myelia	Lipoma with hypertrichosis	Near-total excision	Yes (n/a)	Yes (n/a)	No	1	Alive
2	Mirkin et al., 1990 [34]	F	2	T12-L4	Diastemato-myelia	Lipoma with hypertrichosis	Gross total excision	ARA-C VCR ACT-D DXR	Local + Metastasis (2700 rads)	Yes (Cerebellum)	1.7	Alive
3	Govender et al., 2000 [51]	F	4	T10-Sx	Spina bifida	No	Biopsy only	CSP ETO IFO	Palliative (n/a)	n/a	n/a	n/a
4	Sharma et al., 2005 [57]	F	1.5	L2-L5	Diastemato-myelia	Lipoma with hypertrichosis	Gross total excision	Yes (n/a)	n/a	n/a	n/a	Alive
5	Igbaseimo-kumo et al., 2017 [67]	F	<0.1	L5	Occult dysraphism	Lipoma with hypertrichosis	Gross total excision	VCR ACT-D	No	No	2.5	Alive
6	Our case	M	4	T9-S4	Spina bifida occulta	No	Biopsy only	CPT ETO CYC DXR	Craniospinal (25.5 Gy) + Local (25.5 Gy)	Yes (Local + Lungs)	0.3	Death

Abbreviations: F, female; M, male; yrs., years; n/a, information not available; ARA-C, cytosine arabinoside; VCR, vincristine; ACT-D, actinomycin D; DXR, doxorubicin; CSP, cisplatin; ETO, etoposide; IFO, ifosfamide; CPT, carboplatine; CYC, cyclophosphamide; Gy, gray.
